# Clinical Importance of Binaural Information: Extending Auditory Assessment in Clinical Populations Using a Portable Testing Platform

**DOI:** 10.1044/2021_AJA-20-00168

**Published:** 2021-07-26

**Authors:** Anna C. Diedesch, S. J. Adelaide Bock, Frederick J. Gallun

**Affiliations:** a Department of Communication Sciences and Disorders, Western Washington University, Bellingham; b Communication Sciences & Disorders, Wendell Johnson Speech and Hearing Center, The University of Iowa, Iowa City; c Department of Otolaryngology – Head & Neck Surgery, Oregon Health & Science University, Portland; d VA RR&D National Center for Rehabilitative Auditory Research, VA Portland Health Care SystemVeterans Hospital Road (NCRAR – P5), OR

## Abstract

**Purpose:**

The purpose of this study is to use variability on tests of basic auditory processing to allow identification of those tests that could be used clinically to describe functional hearing ability beyond the pure-tone audiogram and clinical speech-in-noise tests.

**Method:**

Psychoacoustic tests implemented using the Portable Automated Rapid Testing system on a calibrated iPad were evaluated for nine young normal-hearing participants (*M*_age_ = 21.3, *SD* = 2.5) and seven hearing-impaired participants (*M*_age_ = 64.9, *SD* = 13.5). Participants completed 10 psychoacoustic subtests in a quiet room. Correlational analyses were used to compare performance on the psychoacoustic test battery with performance on a clinical speech-in-noise test and with the 4-frequency pure-tone average (4FreqPTA).

**Results:**

Spectral processing ability was highly correlated with 4FreqPTA, and temporal processing ability showed minimal variability across the hearing-impaired group. Tests involving binaural processing captured variability across hearing-impaired listeners not associated with 4FreqPTA or speech-in-noise performance.

**Conclusions:**

Tests that capture the ability to use binaural cues may add information to what current clinical protocols reveal about patients with auditory complaints. Further testing with a larger sample size is needed to confirm the need for binaural measurements and to develop normative data for clinical settings.

Frequently in clinical settings, there are cases where pure-tone hearing thresholds either do not match functional auditory complaints or two individuals with the same pure-tone hearing differ on their performance with hearing aids utilizing the same signal processing algorithms and prescription targets. When pure-tone hearing fails to accurately describe a patient's functional auditory ability, clinicians are able to use speech-in-noise tests in attempting to validate real-world complaints of difficulty hearing in noisy or reverberant situations. However, these complex clinical tests are still much more controlled compared to the patient's real-world environments. Furthermore, a standard diagnostic test battery does not routinely evaluate performance of binaural hearing ability. Instead, clinical speech-in-noise tests are often evaluated either monaurally or diotically over headphones, restricting the patient from utilizing the binaural cues that would be available in standard “cocktail party problem” environments.

Other cues present in realistic environments that are not tested clinically include spectral and temporal modulation (SM and TM), joint spectrotemporal modulation (STM), temporal fine structure (TFS), and grouping cues such as harmonicity, common-onset, and common modulation. Tests of the ability to detect tones in the presence of noise or speech in the presence of intelligible speech maskers are also not commonly used in the clinic and yet may provide useful insight into why people with the similar audiograms vary in terms of their reported auditory abilities. Further testing of basic auditory abilities may be useful when counseling a patient with functional auditory complaints, such as an individual with normal or near-normal pure-tone hearing reporting difficulty communicating in noisy environments. Additional tests may also be useful in refining hearing aid fittings with information beyond pure-tone thresholds and loudness discomfort levels. There are significant issues with extending the current auditory assessment, however, the most obvious being time. Currently, many audiologists have difficulty evaluating the full recommended diagnostic test battery due to short appointment time windows and often have to schedule additional appointments to evaluate further tests of speech-in-noise and other auditory processing tests due to time and potentially space constraints. It is difficult to justify this additional time when there is little consensus on which tests are most informative and few tools available for administering, scoring, or interpreting these tests.

This report is an initial step toward identifying additional tests of basic auditory ability that would have potential utility in terms of being added to diagnostic audiology practice. To ensure that the tests identified have the potential to be used clinically, all were evaluated using the Portable Automated Rapid Testing (PART) application developed at the University of California, Riverside's Brain Games Center (Gallun et al., 2018) that is free and available to the public. The portability of testing would allow audiologists to obtain the information from these additional tests while patients are waiting for the audiologist prior to their diagnostic appointment, immediately following the face-to-face time with their audiologist, or potentially in the future, administered in the comfort of the patients' home. The 10 tests reported here were all described in a recent study by Lelo de Larrea-Mancera et al. (2020), in which normative data were collected for 150 young normal-hearing (NH) listeners. The tests chosen were a small subset of the full capability of what can be tested using a PART system. In order to span the range of tests that could potentially be used clinically but currently are not, the battery tested the ability to detect tones in noise, sensitivity to binaural cues, TFS, SM, TM, STM, and speech-on-speech masking with and without binaural cues.

Tests of tone in noise detection were based on a study by Moore (1987) and were included to allow a rapid measure of frequency selectivity, or the “width” of the putative auditory filter (Patterson, 1976). Common approaches to filter width estimation are either quite time-consuming or rely upon the listener to keep a constant criterion and manually adjust the noise level to provide constant detectability. The method used here provides a rough estimate of filter width by comparing tone in noise detection thresholds with two noises that vary in their masking efficiency as a function of filter width. Auditory filter width at 2 kHz was chosen as it is a critical speech frequency and a location where individuals with mild sloping to moderate amounts of hearing loss tend to have elevated thresholds. Filter width could help explain difference among listeners as those with broader filters will experience more masking from a given noise or other competing sound than will a person with the same threshold but narrower filters. Currently, there are no established clinical tests of filter width and minimal evidence for or against the proposition that such tests could help in fitting hearing aids or counseling patients.

Tests of TFS (Füllgrabe et al., 2015) were chosen to evaluate timing of the auditory nerve firing, which has been shown to correlate with speech-in-noise identification. Füllgrabe et al. (2015) showed that, with audiometrically matched groups, there was an effect of aging and performance on tests of TFS. While effects of aging have been observed, TFS has been shown to be preserved in at least some of those with hearing impairment (Spencer et al., 2016), suggesting that even with damage to cochlear structures, phase locking of the auditory nerve can remain intact for some listeners. Tests of TFS may be good options to show variability across participants with similar pure-tone hearing, particularly if aging is an additional factor. One of the most sensitive ways of measuring TFS is through sensitivity to binaural timing cues (e.g., Grose & Mamo, 2012), but this test cannot distinguish between a TFS deficit or binaural timing deficit. To address this issue, TFS was measured using both monaural and binaural TFS tests and performance was compared to attempt to differentially identify TFS and binaural deficits. Specifically, using the methods of Grose and Mamo (2012), frequency modulation (FM) was used to compare listeners' abilities to use binaural (dichotic FM) and monaural (diotic FM) cue information to assist with FM detection for a low-frequency pure-tone carrier. An additional measure of monaural TFS sensitivity was included by measuring the ability to detect a brief gap inserted between two brief low-frequency tone bursts (Gallun et al., 2014). This stimulus produces both a timing cue, encoded by the timing of spikes on the auditory nerve, and, potentially, a spectral cue, due to small changes in the spread of energy across the basilar membrane. Individuals with reduced temporal processing at the level of the auditory nerve were hypothesized to have elevated thresholds for gap detection and for both types of FM detection. Those with a specifically binaural deficit would be anticipated to have abnormal performance only on the dichotic FM task.

A third set of tests were chosen to evaluate effects of SM, TM, and STM. The auditory nerve adapts rapidly to unmodulated stimuli (e.g., Smith, 1979), and thus complex stimuli, such as modulated signals, are much more effective at driving the central auditory system than are simple signals such as pure tones. One of the most commonly used methods for measuring the sensitivity of the central auditory system is with signals that contain SM and/or TM at low rates, similar to those found in human speech as well as many animal vocalizations (Theunissen & Elie, 2014; Theunissen et al., 2000). By using signals such as these, sensitivity to the acoustical building blocks of speech can be measured without using speech itself, which activates a variety of brain areas that are not responsive to nonspeech signals with the same modulation spectra (Venezia et al., 2019). By evaluating sensitivity to these fundamental acoustical cues, it may be possible to identify auditory processing deficits that arise in brain areas between those sensitive to sound energy, regardless of modulation content, and those brain areas specifically responsive to speech.

The final set of tests evaluated involved a speech corpus called the Coordinated Response Measure (CRM) sentences (Bolia et al., 2000). Though there are time constraints, many clinicians regularly evaluate speech-in-noise testing. To test speech-in-noise performance, clinicians usually use either tests with normative data such as the Quick Speech-in-Noise (QuickSIN) or options such as adding babble or speech noise stimuli to clinical word recognition tests. The difference between these tests and those used here, based on the CRM and originally developed by Marrone et al. (2008), were twofold. First, the CRM has a fixed sentence structure with a call sign and two key words (one color and one number), making it difficult to tell which of two CRM sentences is the target without connecting the call sign to the keywords. This “informational masking” is rarely tested clinically and may be more closely related to auditory complaints than is speech in noise or speech in babble, where performance is based primarily on the audibility of the target words rather than the ability to form accurate streams across time for the competing sentences as in the CRM tasks. The second way in which these tasks differ from tests such as the QuickSIN is that two conditions are compared, as in the diotic and dichotic FM tasks described in the TFS testing section. In one condition, the target and the two masking sentences are “colocated,” which means that the target and distractor talkers are located directly in front of the listener at 0° azimuth. In the comparison condition, the target is still at 0°, but the two competing CRM sentences are spatially separated from the target to the left and the right by ± 45°. Spatial release from masking (SRM) is defined as any change in speech recognition performance between these two conditions. Variability in performance within and across conditions, as well as in SRM, is hypothesized to relate to the ability to use spatial and spectrotemporal cues to distinguish the target from the masking talkers (Gallun et al., 2013).

Tests of auditory filter width, temporal fine-structure, binaural sensitivity, complex modulated signals, and binaural speech-on-speech masking will add to the diagnostic test battery currently administered in the clinic. However, while it is feasible to complete this testing on clinical patients in a laboratory setting, it is not feasible to expect audiologists to administer all of these tests to their clinical patients. Here, variability on tests of basic auditory processing used in Lelo de Larrea-Mancera et al. (2020) will be evaluated to identify which of those tests could be used clinically to describe functional hearing ability beyond the pure-tone audiogram and clinical speech-in-noise tests. These tests could be added to routine clinical procedures to fill in the gaps of functional auditory complaints and pure-tone hearing thresholds measured in the clinic. Results from 150 NH listeners who participated in Lelo de Larrea-Mancera et al.'s (2020) experiment were used as normative data. Results from a small group of hearing-impaired (HI) participants suggest that the binaural measures are the most likely to add information distinct from the audiogram to the clinical test battery, but all of the tests in the battery showed potential promises for understanding the ways in which the listeners differed one from another.

## Method

### Participants

Nine young NH and nine participants with mild-to-moderate sensorineural hearing loss were recruited. Two HI participants were excluded due to missing audiometric data, and a second run on the experimental protocol, one HI participant (HI8) and the other HI participant (HI9) failed a cognitive screening. Data were analyzed for 16 participants: nine NH participants (*M*_age_ = 21.3, *SD* = 2.5, two males) and seven HI participants (*M*_age_ = 64.9, *SD* = 13.5, two males). HI8 and HI9 were excluded from the analyses. NH participants were included as a comparison to normative data collected by Lelo de Larrea-Mancera et al. (2020).

**Figure 1.**
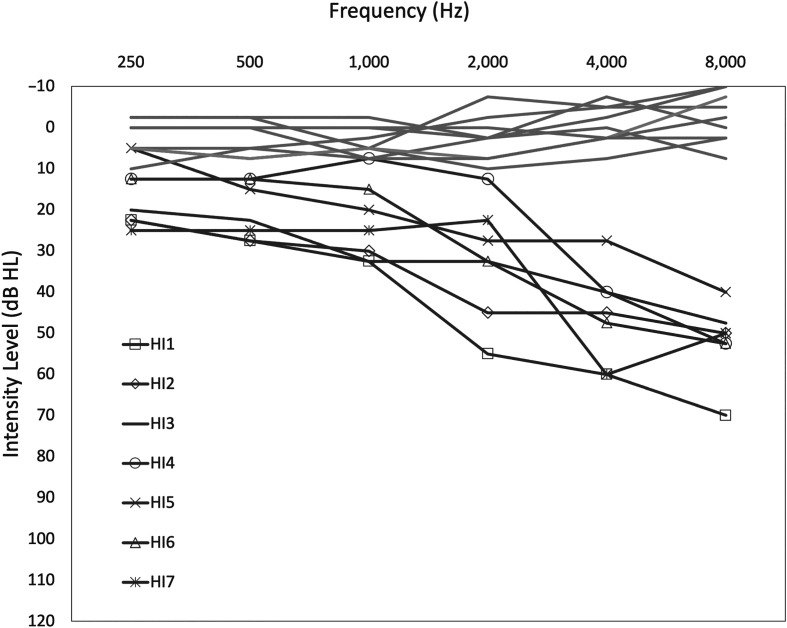
Gray lines represent each individual listener's pure-tone hearing at octave frequencies (250–8000 Hz) averaged across ears. Light gray lines represent the normal-hearing participants, and dark gray lines with symbols represent the hearing-impaired listeners. Across ear differences were limited to 10 dB HL. Hearing-impaired listeners' pure-tone thresholds were limited to mild-to-moderate amounts of sensorineural hearing loss. Refer to Table 1 for individual ear pure-tone averages. HI = hearing impaired.

Figure 1 displays average pure-tone hearing thresholds across both ears for each research participant. One of the seven HI participants (HI7) completed only one run of the experimental protocol, and one subtest was unable to be completed by one HI participant (HI1) due to audibility issues. Missing data from participants HI7 and HI8 were related to university closures due to COVID-19, and HI8 was excluded from the study because audiometric data were not available to complete the data analysis.

All participants were screened for peripheral auditory function and cognition prior to experimental testing. Otoscopy, tympanometry, pure-tone hearing thresholds, and a test of cognition were evaluated to determine eligibility. Participants were excluded from the study if they scored lower than 26 points on the Montreal Cognitive Assessment, had greater than a mild-to-moderate sloping sensorineural hearing loss, showed signs of a conductive hearing loss, or had thresholds that differed by greater than 10 dB across ears for octave frequencies between 250 and 8,000 Hz. After screening and audiometric testing, participants were then evaluated for performance on a clinical speech-in-noise test using the QuickSIN. Two sentence lists were evaluated in each ear. For statistical analysis, hearing was operationalized by the 4-frequency pure-tone average (4FreqPTA), using an average of 500-, 1000-, 2000-, and 4000-Hz thresholds from both ears. Four thousand hertz was included in the pure-tone average to better account for the differences in HI participants with mild-to-moderate gently sloping sensorineural hearing loss, several of whom had normal low-frequency thresholds (see Figure 1). Demographic details of participants are available in Table 1, including 4FreqPTA and performance on the QuickSIN.

**Table 1.** Gender, age, average audiometric thresholds, and average QuickSIN results are displayed for each research participant.

ID	Gender	Age	4FreqPTA (dB HL)			QuickSIN (dB SNR loss)		
			LE	RE	Average	LE	RE	Average
**HI1**	**F**	**83**	**46.25**	**41.25**	**43.75**	**4**	**6**	**5**
HI2	F	59	37.50	36.25	36.88	4.5	4	4.25
HI3	F	71	28.75	35.00	31.88	1.5	5	3.25
**HI4**	**M**	**70**	**20.00**	**16.25**	**18.13**	**4**	**7**	**5.5**
**HI5**	**F**	**39**	**23.75**	**21.25**	**22.50**	**2**	**0.5**	**1.25**
HI6	F	66	28.75	25.00	26.88	2.5	1.5	2
HI7	M	66	33.75	32.00	33.13	2.5	3	2.75
HI, *M* (*SD*)		64.9 (13.5)	31.3 (8.8)	29.6 (9)	30.4 (8.7)	3 (1.2)	3.9 (2.4)	3.4 (1.5)
NH1	F	26	5.00	2.50	3.75	0	1	0.5
NH2	F	19	−5.00	0	−2.50	2	2	2
NH3	F	20	0	0	0	2.5	2.5	2.5
NH4	F	20	7.50	7.5	7.50	1.5	2	1.75
NH5	F	23	2.50	−1.25	0.63	1	2.5	1.75
NH6	F	23	−2.50	−2.50	−2.50	0.5	1.5	1
NH7	F	20	−1.25	1.25	0	1	0.5	0.75
NH8	M	18	3.75	7.50	5.63	1.5	1.5	1.5
NH9	M	23	2.50	6.25	4.38	1	0.5	0.75
NH, *M* (*SD*)		21.3 (2.5)	1.4 (3.9)	2.4 (3.8)	1.9 (3.6)	1.2 (0.8)	1.6 (0.8)	1.4 (0.7)
All, *M* (*SD*)		40.4 (24.0)	14.5 (16.5)	14.3 (15.3)	14.4 (15.9)	2 (1.3)	2.6 (2.0)	2.3 (1.5)

*Note.* The upper rows show data for the seven hearing-impaired (HI) participants and the mean (*SD*) for the HI group. The lower rows show data for the nine normal-hearing (NH) participants and the mean (*SD*) for the NH group. All mean (*SD*) shows mean (*SD*) for both groups combined. Pure-tone averages (PTAs) were calculated using a 4-frequency PTA (4FreqPTA) for 500-, 1000-, 2000-, and 4000-Hz thresholds and are displayed for the left and right ears (LE, RE) and an average of both. QuickSIN results are an average of two test lists for each ear and the average of four test lists from both left and right ears. Three research participants (HI1, HI4, and HI5 in **bold**) are referenced in the Discussion section. QuickSIN = Quick Speech-in-Noise Test.

Upon completion of the clinical tests, the remaining tests were all completed in a quiet room using PART. The PART test battery chosen was designed by Lelo de Larrea-Mancera et al. (2020) and used to evaluate reliability of the platform and the method of testing on 150 undergraduate students from University of California, Riverside. Lelo de Larrea-Mancera et al., using an identical system with the same strongly attenuating headphones, compared performance in a sound booth and in a room with recorded cafeteria noise and found no statistically reliable difference. This supports testing the PART battery in a quiet room rather than in a sound booth. Two repetitions of the 10 PART subtests were completed for all but one participant (HI7). Testing was conducted over two sessions lasting approximately 2 hr each.

HI participants were recruited from the Western Washington University Speech-Language-Hearing Clinic, and NH participants were recruited via word of mouth. All recruitment and testing procedures were in compliance with and approved by Western Washington University's Institutional Review Board for Human Subjects Research. All participants were compensated $15 an hour for their time in the form of a gift card to a local grocery store.

### Equipment

To evaluate participants' peripheral auditory function, otoscopy, tympanometry, and pure-tone hearing testing were accomplished prior to testing. Tympanometry was conducted on a Grason-Stadler Inc. (GSI) TympStar platform. Pure-tone audiometry was evaluated at octave frequencies between 250 and 8000 Hz in a sound booth using a GSI 61 audiometer and Etymotic Research ER-3 insert earphones.

Psychoacoustic tests using PART were presented using a 10.5-in. iPad Pro using Sennheiser 280 Pro headphones, which have over 20 dB of passive attenuation across the frequencies included in the stimuli tested here. Calibration was accomplished at the National Center for Rehabilitative Auditory Research's anechoic chamber located in Portland, Oregon. Measurements were made on a Brüel & Kjær Head and Torso Simulator with the iPad volume set to maximum output. Calibration adjustments were made using the internal PART calibration system. See Gallun et al. (2018) for further detail on the acoustic validation of PART.

### PART Procedures and Stimuli

PART subtests were chosen to evaluate basic auditory processing ability beyond the traditional diagnostic audiology test battery. The 10 subtests used by Lelo de Larrea-Mancera et al. (2020) were tested in the following order: tone in noise (two noise conditions); FM (two conditions); gap detection; CRM sentence tests; and TM, SM, and STM. The tests are described briefly below. For further details, see Lelo de Larrea-Mancera et al.

Tone in noise (Moore, 1987): The ability to detect a tone in two noise maskers as adapted from Moore (1987) was tested as in Lelo de Larrea-Mancera et al. (2020). In addition, a metric of filter width was added to allow comparisons among listeners and across studies. Thresholds were estimated by presenting a 2-kHz tone at 45 dB SPL for 500 ms, either in one spectrally continuous narrowband noise (1.6–2.4 kHz; “no-notch condition”) or in a spectrally silent gap between two narrowband noises (1.2–1.6 kHz and 2.4–2.8 kHz; “notch condition”). Estimates of filter width were accomplished by subtracting threshold in the notch condition from threshold in the no-notch condition. A value of zero would indicate no difference in threshold with the introduction of an 800-Hz wide spectral notch, thus suggesting that the putative auditory filter at 2 kHz was so wide that all of the noise energy fell inside the filter for both notch and no-notch stimuli. Lower values indicate narrower filters, with a difference of −24 dB being the published value for experienced young listeners with NH (Patterson, 1976). The method used here, with naïve listeners, is expected to produce an average value of −19 dB based on the supplemental data set provided by Lelo de Larrea-Mancera et al. The level of the 2-kHz target tone can be adjusted to ensure audibility in PART settings but was not done so in this experiment. The level of 45 dB SPL was audible to all but one participant (HI1).

Thresholds were measured by adaptively varying the noise level using a 4-interval, 2-alternative forced choice (4I-2AFC) two-down, one-up adaptive tracking procedure. On each trial, the participant was presented with four intervals marked by virtual buttons that are shown on the iPad and that change color one by one as a sound is played. The first and last buttons are always presented with a standard sound, while the second and third buttons contain either a standard or the target sound. The target differs in the parameter to be evaluated, such as modulation depth or noise level. The observer is forced to choose either the second or third button, and when the target is correctly identified in two successive trials, the parameter value is changed to make the task more difficult, such as by decreasing the modulation depth or increasing the noise level. When an incorrect response is given, the parameter value is changed such that the task is easier to perform. Every time the parameter value “reverses” from getting easier to harder or harder to easier, the value at which this reversal occurs is recorded. Once three reversals have occurred, the size of the parameter changes is decreased and the average of the next six reversals is taken as the threshold. Linear steps were taken for the Tone-in-Noise; Spectral, Temporal, and Spectrotemporal subtests; and logarithmic steps for the FM and Temporal Gap Detection subtests. The only subtest that deviated from the 4I-2AFC adaptive track procedure was the SRM, where a progressive track was utilized.

**Figure 2.**
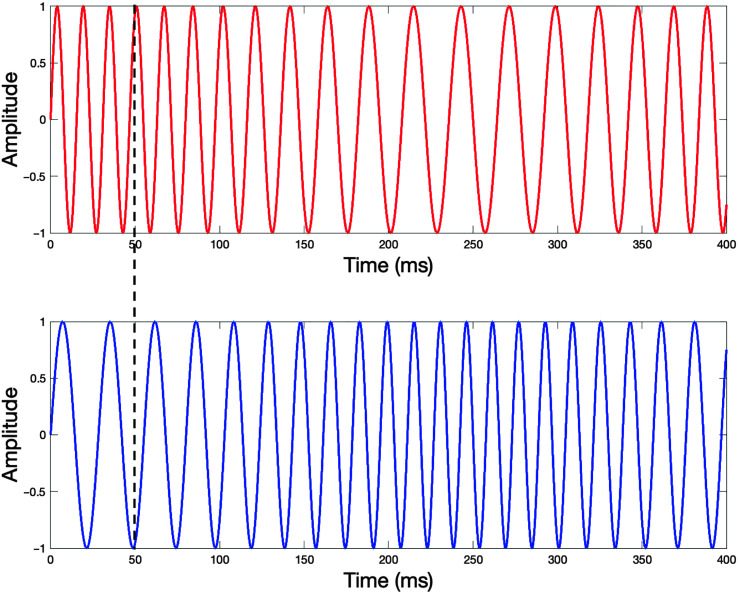
A diagram of a dichotic frequency modulation stimulus waveform is displayed, with the red waveform (top) indicating the signal presented to the right ear and the blue waveform (bottom) indicating the waveform presented to the left ear. Frequency modulations move in a different manner for each ear. Here, the right ear depicts a higher frequency modulating to a lower frequency while the left ear has a lower frequency modulated to a higher frequency. The black dashed line across both waveforms displays the difference in phase across the two ears, where around 50 ms in this example, the two stimuli are at opposite phase, displaying a strong interaural phase difference in the temporal fine structure of this dichotic stimulus. The diotic FM stimulus (not displayed) would consist of the same waveform in both, thus eliminating the interaural phase differences. The standard stimulus for both diotic and dichotic FM tasks contains a diotic signal that does not modulate in frequency over time. Note that for illustration purposes, the carrier frequency is 50 Hz rather than 500 Hz and the modulation depth is 18 Hz. The modulation rate of 2 Hz is the same as in the stimuli presented in the experiment described in the text. FM = frequency modulation.

FM (Grose & Mamo, 2012; Hoover et al., 2019; Whiteford et al., 2017; Whiteford & Oxenham, 2015): Diotic and dichotic FM stimuli were presented at 75 dB SPL for 400 ms. The standard condition included identical pure tones presented in phase to both ears (“diotic”), with a carrier randomized between 460 and 550 Hz. In the “diotic FM” condition, the target was frequency modulated (FM) at a rate of 2 Hz, but was identical in the two ears. In the “dichotic FM” condition, the FM applied to the target was inverted at one ear, producing an interaural phase difference (IPD). During diotic presentation, FM must be presented at greater depths to be detectable compared to the dichotic condition. This is due to the additional IPD cue in the dichotic condition, as observed in the example waveforms shown in Figure 2. In the dichotic presentation, FMs go from low to high in one ear and high to low in the other ear. Diotic presentation utilizes the same stimulus but with the same modulation pattern received in both ears, making the task more difficult and providing no IPD cue. Figure 2 depicts an example dichotic FM stimulus generated with a 50-Hz carrier frequency and an 18-Hz modulation depth in order to illustrate these phenomena, which are difficult to visually identify in the actual stimuli used. The same four-interval adaptive tracking procedure was used as in the tone in noise tasks, but in this case, the adaptive parameter was modulation depth. In both conditions, and in every interval, the stimulus carrier frequency was randomly selected from a flat distribution between 460 and 550 Hz. This randomization ensured that listeners were required to track the changes in frequency across time rather than simply comparing the beginning or ending frequencies and choosing the interval in which this frequency was different.

Gap detection (Gallun et al., 2014): In the Temporal Gap test, on each interval, listeners were presented with two, 4-ms 500-Hz tone bursts presented diotically at 80 dB SPL. In the standard interval, the two bursts were contiguous, while in the target interval, a silent gap was introduced between them.

**Figure 3.**
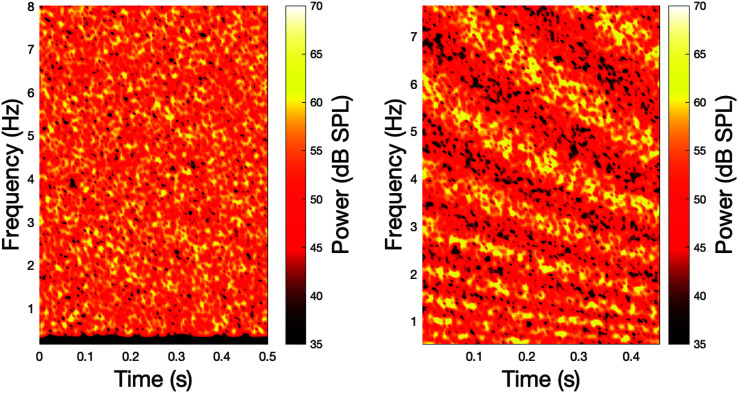
For the temporal, spectral, and spectrotemporal tasks, complex auditory stimuli are used. Here, spectrograms for the standard (left panel) and target spectrotemporal spectrogram (right panel) are displayed. The standard stimuli randomly varies in frequency and intensity over time, thus creating a standard noise. Here, the target for the spectrotemporal task shows intensity variations in frequency and amplitude modulations over time. In the temporal task (not displayed), frequency is fixed and amplitude modulates over time (differences in intensity would be observed as vertical striations in the spectrogram). In the spectral task, amplitude over time is fixed and frequency would modulate over time (differences in intensity would be observed as horizontal striations in the spectrogram).

SM, TM, and STM: Each of these tests requires the listener to distinguish an unmodulated noise ranging from 0.4 to 8 kHz from the same noise modulated spectrally at 2 cycles/octave, temporally at 4 Hz, or spectrotemporally at both 2 cycles/octave and 4 Hz. Spectrograms of the standard stimulus and a spectrotemporally modulated stimulus can be observed in Figure 3. These three conditions were modulated either by spectral cues (*x*-axis), temporal cues (*y*-axis), or both over the adaptive track with modulation depth in dB as the adaptive parameter.

**Figure 4.**
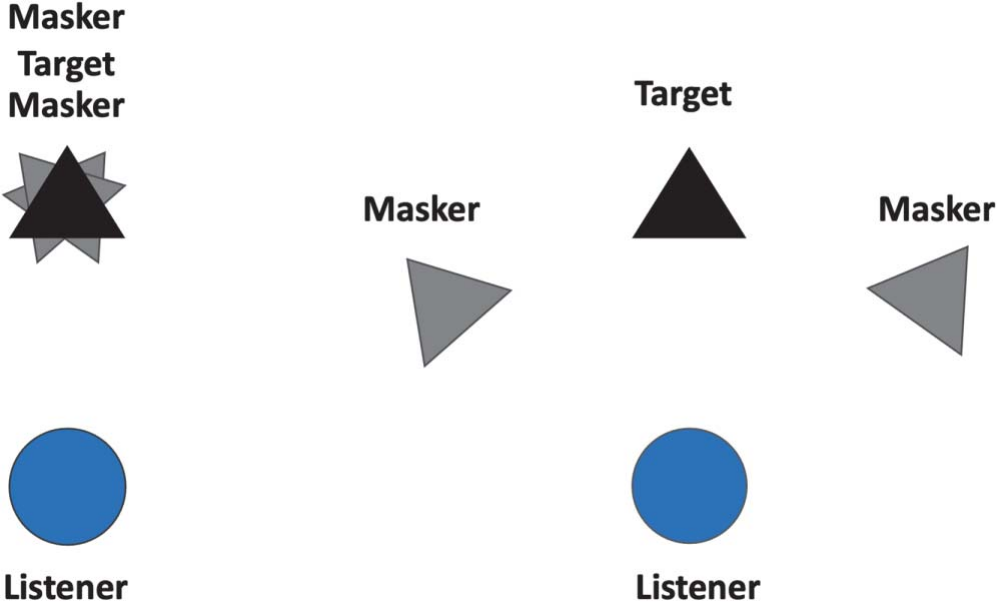
Methods for spatial release from masking are depicted. The listener on the left is performing a colocated condition where the target and maskers are all at 0° azimuth, while the listener on the right is performing the spatially separated condition where the target is at 0° and maskers are at ± 45°.

Colocated and spatially separated SRM: In this task, the listener was required to identify the color and number keywords of a target talker CRM sentence in the presence of two competing CRM sentences, all of which had unique call signs and keywords. The target sentence was always identifiable by the use of the call sign “Charlie.” Both target talker (always presented at 0° azimuth) and two masker talkers (0° for colocated condition, ± 45° for spatially separated condition) used male speakers (see Figure 4 for a depiction of the speaker setup). Spatially separated masker sentences contained spatial cues in comparison to the colocated maskers all presented to the front of the listener. Target sentences were fixed at a root-mean-square level of 65 dB SPL, and masker level was progressively varied using the progressive tracking algorithm developed by Gallun et al. (2013). Participants were instructed to listen for the color and number combination from the talker using the sentence “Ready CHARLIE go to COLOR NUMBER now,” while the distractor talkers used the same sentence structure with one of the other seven call signs (such as “Ringo” and “Baron”). For this subtest, rather than a four-interval task with buttons that changed color, participants were shown a grid of four colors and eight numbers. All SRM data are displayed in units of target-to-masker ratio (TMR) where the target level (65 dB SPL) is subtracted from the masker level. Masker levels began at a TMR of −10 dB (55 dB SPL) and progressively adapted in two dB steps to a + 8 dB (73 dB SPL). Two sentences were presented at each TMR, for a total of 20 trials, and threshold was calculated by subtracting the number of correct responses from 10. Thus, perfect performance results in a TMR estimate of 10–20 = −10 dB, while no correct responses result in a TMR estimate of 10–0 = 10 dB. Gallun et al. (2013) showed that this method provides a reliable, but slightly biased, estimate of 50% correct performance. Estimates of threshold masker level in dB SPL can be obtained by subtracting the threshold TMR from the target level of 65 dB SPL. Thus, −10-dB TMR is equivalent to a masker level of 65 – (−10) = 75 dB SPL.

## Results

Thresholds from the 10 experimental subtests were compared separately to pure-tone hearing thresholds and performance on a clinical speech-in-noise test. Considering that two individuals with similar pure-tone hearing may perform differently on functional tests of hearing (e.g., speech-in-noise), the goal was to identify specific subtests within the larger test battery that show variability across participants with similar clinical thresholds. Tests showing variability in responses for participants with similar pure-tone hearing thresholds would be viewed as potential candidates for informative additions to the clinical diagnostic test battery. To explore this question, thresholds were correlated with pure-tone hearing and performance on the QuickSIN.

### Comparisons With Published Data

**Figure 5.**
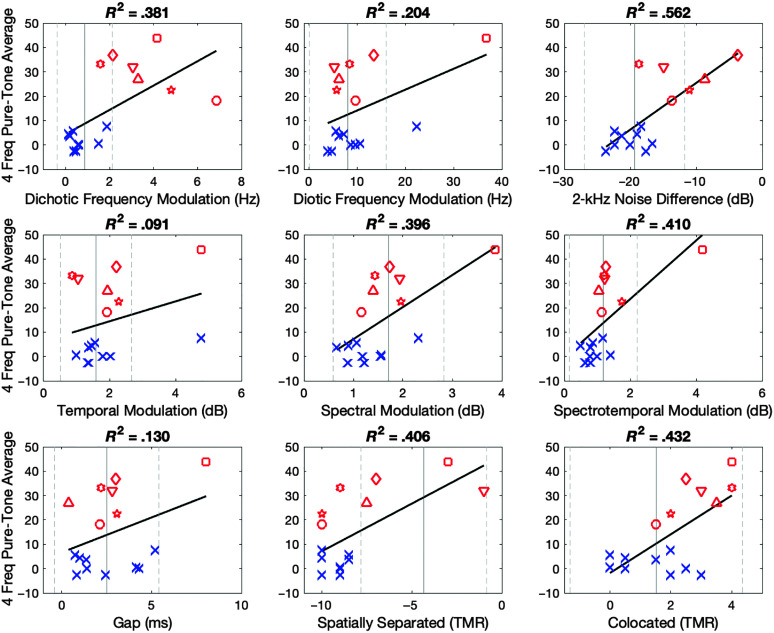
Subplots represent 4-frequency pure-tone average (4FreqPTA) correlated with thresholds for each experimental subtest, with the exception of 2-kHz Noise Difference subtest, which was derived by taking NoNotch – Notch. Blue x*s* represent normal-hearing participants, and red open symbols represent hearing-impaired participants. Black linear regression line and *R* ^2^ values were derived for performance across all participants. A vertical solid gray line represents mean thresholds from normative data from Lelo de Larrea-Mancera et al. (2020), with gray vertical dashed lines representing ± 1 *SD*. Only 6 HI participants completed the 2-kHz Notch Noise subtests due to audibility at 2 kHz for HI1. HI Subject key: HI1 (square), HI2 (diamond), HI3 (downward triangle), HI4 (circle), HI5 (5-point star), HI6 (triangle), HI7 (6-point star). TMR = target-to-masker ratio; HI = hearing-impaired.

Table 2 lists the measured thresholds on the 10 tests. Listener HI1 was the only one unable to provide thresholds for all of the measures. As can be seen in Table 1, HI1 was the oldest participant, at 83 years, and had the highest 4FreqPTA, at 43.75 dB HL. The data for all participants are plotted as a function of 4FreqPTA in Figure 5, with the HI participants marked with consistent symbols across panels. In nearly every case, HI1 has the score most different from the mean of the data reported by Lelo de Larrea-Mancera et al. (2020), which is indicated by a solid vertical line. The values 1 *SD* above and below the mean, calculated based on the supplemental data provided with that publication, are marked with vertical dashed lines.

**Table 2.** Thresholds for PART subtests are displayed for each HI and NH participants.

ID	Dichotic FM	Diotic FM	2-kHz No-Notch	2-kHz Notch	Temporal	Spectral	Spectrotemporal	Gap	Separated SRM	Colocated SRM
**HI1**	**4.17**	**36.82**	**x**	**x**	**4.79**	**3.87**	**4.18**	**8.03**	**−3**	**4**
HI2	2.14	13.46	47	50.67	2.20	1.74	1.27	3.00	−7	2.5
HI3	3.06	5.25	51	66	1.05	1.93	1.23	2.82	−1	3
**HI4**	**6.85**	**9.65**	**57.98**	**71.67**	**1.92**	**1.17**	**1.13**	**2.12**	**−10**	**1.5**
**HI5**	**4.80**	**5.73**	**55.67**	**66.67**	**2.28**	**1.95**	**1.74**	**3.09**	**−10**	**2**
HI6	3.29	6.22	51.66	60.33	1.93	1.40	1.05	0.38	−7.5	3.5
HI7	1.59	8.49	55.66	74.33	0.86	1.43	1.20	2.20	−9	4
HI, *M* (*SD*)	3.7 (1.8)	12.2 (11.2)	53.2 (4.0)	64.9 (8.5)	2.1 (1.3)	1.9 (0.9)	1.7 (1.1)	3.1 (2.4)	−6.8 (3.5)	2.9 (1.0)
NH1	0.19	6.28	55.33	76.67	1.37	0.67	0.80	1.37	−8.5	1.5
NH2	0.37	3.94	54.67	78.33	1.35	1.22	0.80	0.85	−10	2
NH3	0.57	9.74	59	79	1.8	1.19	0.78	4.31	−9	2.5
NH4	1.89	22.34	58.33	76.67	4.79	2.32	1.17	5.19	−10	2
NH5	1.51	10.63	56.33	73	0.99	1.57	1.40	4.15	−9	0
NH6	0.48	4.75	56.67	74.33	1.37	0.88	0.63	2.45	−9	3
NH7	0.62	8.65	55	77.33	2.03	1.55	0.98	1.42	−9	0.5
NH8	0.35	5.67	57.33	79.67	1.57	1.07	0.87	0.76	−8.5	0
NH9	0.15	7.24	58	77	1.45	0.90	0.50	1.00	−10	0.5
NH, *M* (*SD*)	0.7 (0.6)	8.8 (5.5)	56.7 (1.5)	76.9 (2.1)	1.9 (1.1)	1.3 (0.5)	0.9 (0.3)	2.4 (1.7)	−9.2 (0.6)	1.3 (1.1)
All, *M* (*SD*)	2.0 (2.0)	10.3 (8.4)	55.3 (3.2)	72.1 (8.1)	2.0 (1.2)	1.6 (0.8)	1.2 (0.8)	2.7 (2.0)	−8.2 (2.6)	2.0 (1.3)

*Note.* Three HI participants' data are in **bold** and are referenced in the Discussion section. Spatial release from masking (SRM) subtests (Spatially Separated and Colocated) are displayed in target-to-masker ratio units, derived by subtracting masked thresholds from the target level (65 dB). All other threshold values were taken directly from the PART test battery standard threshold output. Mean (*SD*) data are shown for NH and HI groups separately and combined in the All *M* (*SD*) row. PART = Portable Automated Rapid Testing; HI = hearing-impaired; NH = normal-hearing; FM = frequency modulation.

The tone in noise measures were similar to the published data, with all of the NH participants producing noise masker thresholds within 2.5 dB of the published values of 57 dB SPL for the no-notch condition and with 5 dB of the published value of 76 dB SPL for the notched noise condition. Four of the NH listeners even produced differences in the range of the 24 dB reported by Patterson (1976) for four listeners with extensive psychoacoustic training. Although Lelo de Larrea-Mancera et al. (2020) did not report the statistics for the difference between the notch and no-notch threshold, the supplemental data were used to do so, resulting in a value of −19 dB, which is within 5 dB of the values observed for each of the NH listeners. The majority of the HI listeners had difference values that were more than 5 dB closer to zero than the mean of the published data, and the notched noise threshold values were at least 10 dB lower than the published mean for four of the six HI participants who could perform the task.

Dichotic FM thresholds were within 1 Hz of the published mean value of 0.89 Hz for all of the NH participants but were higher by 2 Hz or more above the mean for all but two of the HI participants. Diotic FM, on the other hand, was within 5 Hz of the published mean for all but two of the participants: one from the HI group and the other from the NH group.

Published means for TM, SM, and STM thresholds were 1.6 dB, 1.7 dB, and 1.2 dB, respectively. One NH participant and one HI participant produced thresholds greater than 1 dB above these values. Similarly, only one participant (HI1) produced gap thresholds more than 3 ms greater than the published mean of 2.5 ms. As for the speech-on-speech masking, none of the participants in the colocated SRM condition required TMR values more than 3 dB greater than the published mean of 1.5-dB TMR, and all but two of the HI participants were able to perform the separated condition at TMR values at least 2 dB below the published mean.

### Correlations With Pure-Tone Average

To better understand the variability in performance across listeners, thresholds for each subtest were correlated with participants' 4FreqPTA. Linear regressions and *R* ^2^ values for each subplot in Figure 5 represent the regression line and variance for all participants, NH and HI. NH data are represented by blue x's, and individual symbols have been assigned for each HI participant, plotted in red. Vertical lines represent the mean (solid line) and ± 1 *SD* (dashed lines) based on the normative data from Lelo de Larrea-Mancera et al. (2020). Raw data for each participant are presented in Tables 1 (demographics) and 2 (PART subtests). Without correction, *R* ^2^ values of .25 are significant at the *p* = .05 level. Here, a correction for multiple comparisons was used making *R* ^2^ values of .5 significant at the *p* = .05 level. Correlations for each test comparison are displayed in Table 3, with *R* ^2^ values of .5 and above shown in bold.

**Table 3.** *R* ^2^ values across tests.

Variable	4FreqPTA	QuickSIN	Age	DichFM	DioFM	2 kHz	TM	SM	STM	Gap	Sep	Col
**4FreqPTA**		.467	**.848**	.381	.204	.**562**	.091	.396	.410	.130	.406	.432
**QuickSIN**			.**614**	.488	.256	.266	.095	.257	.299	.213	.201	.196
**Age**				.**533**	.137	.447	.033	.265	.336	.078	.401	.450
**Dichotic FM**					.089	.373	.105	.208	.247	.093	.055	.096
**Diotic FM**						.033	.**754**	.**754**	.**703**	.**748**	.131	.100
**2**-**kHz Notch Difference**							.024	.160	.298	.027	.078	.149
**Temporal Modulation (TM)**								.**607**	.433	.**504**	.026	.060
**Spectral Modulation (SM)**									.**842**	.**680**	.318	.181
**Spectrotemporal Modulation (STM)**										.**607**	.294	.171
**Gap**											.149	.118
**Separated TMR**												.215
**Colocated TMR**												

*Note.* 4FreqPTA and QuickSIN values are an average of performance for both ears. Two-kHz Notch Difference represents the difference between No-Notch and Notch conditions. Estimates of shared variance greater than .5 are in **bold**. 4FreqPTA = 4-frequency pure-tone average; QuickSIN = Quick Speech-in-Noise Test; DichFM = Dichotic FM; DioFM = Diotic FM; Sep = Separated TMR; Col = Colocated TMR; TMR = target-to-masker ratio.

Strong correlations with 4FreqPTA are shown for 2-kHz Noise Threshold Difference (*R* ^2^ = .562), SM (*R* ^2^ = .396), STM (*R* ^2^ = .410), dichotic FM (*R* ^2^ = .381), and both SRM subtests (Spatially Separated, *R* ^2^ = .406; Colocated, *R* ^2^ = .432). On the other hand, TM (*R* ^2^ = .091) and gap detection (*R* ^2^ = .130) showed poor correlation with pure-tone hearing. Variability in responses across similar 4FreqPTAs was seen most prominently for dichotic FM and the SRM tests for HI listeners; however, NH listeners performed quite similarly in Dichotic FM and Spatially Separated SRM subtests. Several participants' data were outside ± 1 *SD* for Dichotic FM and Spatially Separated SRM subtests. Specifically, most HI participants perform worse on the Dichotic FM subtest and both NH and some HI listeners perform better in the spatially separated SRM. Variability in thresholds across PART subtests was seen for both groups in the Colocated SRM subtest, and some variability was seen in both groups for the Gap Detection and Temporal subtests. Variability in performance for only the HI group was also seen for dichotic FM and there were consistently one or two outliers in performance across the PART subtests.

### Correlation With QuickSIN

**Figure 6.**
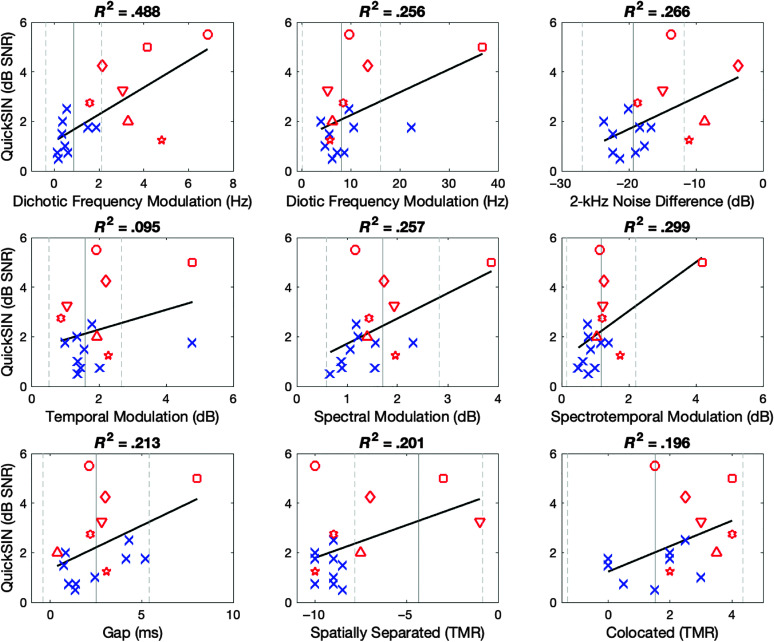
Subplots represent average QuickSIN scores across both ears (individual ear scores are listed in Table 1) correlated with thresholds for each experimental subtest, with the exception of 2-kHz Noise Difference being derived from Notch and No-Notch subtests. Participant symbols and gray lines indicating normative data are the same as depicted in Figure 1. Black linear regression lines and *R* ^2^ values were calculated across all normal-hearing and hearing-impaired listeners. HI Subject key: HI1 (square), HI2 (diamond), HI3 (downward triangle), HI4 (circle), HI5 (5-point star), HI6 (triangle), HI7 (6-point star). QuickSIN = Quick Speech-in-Noise Test; HI = hearing-impaired.

To better understand the potential relationships between auditory processing abilities and the ability to understand speech in complex environments, performance on the experimental tests was correlated with performance on the QuickSIN, a common clinical test of speech in noise. In Figure 6, relationships are shown for QuickSIN thresholds and the auditory processing tests, with linear regression lines and *R* ^2^ values calculated for both NH and HI groups combined. Symbols and layout are the same as Figure 5. Thresholds for each subtest are listed by research participant in Tables 1 and 2.

The subtest most correlated with QuickSIN thresholds was dichotic FM (*R* ^2^ = .488). Other subtests that correlated well with the QuickSIN were the 2-kHz Noise Threshold Difference (*R* ^2^ = .266), diotic FM (*R* ^2^ = .256), spectral processing (*R* ^2^ = .257), and spectrotemporal processing (*R* ^2^ = .299). The subtest showing the weakest correlation was temporal processing (*R* ^2^ = .095), similar to comparisons with 4FreqPTA. Subtests showing moderate relationships included the tests of SRM (Spatially Separated *R* ^2^ = .201, Colocated *R* ^2^ = .196) and Gap Detection (*R* ^2^ = .213).

Substantial within-group variability for the HI participants was observed for Dichotic FM and SRM subtests. For the NH listeners, variability was seen for the Colocated SRM subtest and Gap Detection.

## Discussion

Current clinical best practice protocols evaluate pure-tone hearing thresholds, speech reception thresholds, and word recognition in quiet, among other behavioral and objective tests. Often, audiologists will include a test of speech in noise, time permitting, if patient complaints pertain to difficulty hearing in noisy situations. However, if a patient has either normal or near-normal pure-tone hearing or their hearing is not bad enough to prescribe hearing aids, then a patient with functional auditory complaints may receive counseling that they are clinically “normal” and that hearing aids will likely not benefit the patient. Additionally, individuals with similar pure-tone hearing thresholds may differ on functional tests, such as speech-in-noise tests (e.g., QuickSIN). The objective of this research is to identify auditory tests not regularly administered in the clinic that may help fill in the gaps of performance on routine clinical tests and functional auditory complaints.

One way to fill in the gaps would be to observe variability in performance for individuals with similar clinical test results, for example, differences in performance on the QuickSIN for individuals with similar pure-tone hearing. Comparisons to speech-in-noise performance are already available to audiologists who routinely evaluate tests such as QuickSIN in their clinical practice. Here, two pairs of research participants were shown to have differences across their QuickSIN and 4FreqPTA results. For instance, HI4 and HI5 have similar pure-tone hearing but have the best and worst scores on the QuickSIN among HI participants in this data set. Furthermore, HI1 and HI4 performed similarly poor on the QuickSIN despite having the lowest and highest 4FreqPTA in the HI group. Aging could be a confounding variable for these differences, particularly for HI4 (70 years old) and HI5 (39 years old); however, additional tests evaluating auditory processing ability may be able to better describe the difference for individuals with similar pure-tone hearing.

The differences in functional performance, such as those seen with participants HI1, HI4, and HI5, suggest that further testing is needed to obtain a more complete picture of patients' basic auditory ability. Previous research has attempted to relate different psychoacoustic measures of auditory function to speech recognition for HI listeners (Bernstein et al., 2013; Strelcyk & Dau, 2009), and the data seen here are consistent with past findings. However, the attempts made here are more efficient than classic psychoacoustic methods, making it feasible for a clinician to quickly obtain similar spectrotemporal and TFS thresholds for comparison.

Another issue faced in current clinical practice is a lack of binaural testing. Despite the fact that the majority of clinical patients utilize binaural processing in the real world, nearly all routine clinical tests are accomplished with monaural presentation. Here, the two tests indicating the greatest variability across research participants, when evaluating both correlations as well as visual spread for HI participants in Figures 5 and 6, involved binaural hearing: Dichotic FM and SRM. While clinicians may not have time to add all tests of spectral, temporal, spectrotemporal, and temporal fine-structure processing ability to their current test battery, the addition of Dichotic FM and SRM may be two tests that could compliment current clinical practice by providing additional information about listeners' basic auditory ability. Furthermore, additional tests, such as Dichotic FM and SRM, may confirm auditory complaints from individuals with normal pure-tone hearing thresholds such as HI4 and HI5. However, a lack of reimbursement for additional testing continues to be an issue in clinical practice. Plus, due to time constraints, audiologists may find it difficult to complete the current diagnostic test battery according to best practice guidelines, let alone add additional tests unless further testing is critical for patient care.

A solution to administering additional auditory tests would be the ability to evaluate patients on a portable platform, such as in a kiosk, or on a portable device such as a tablet with calibrated headphones. Here, we evaluated patients in a quiet room using a calibrated iPad and over-the-ear headphones. The entire experimental protocol was completed in approximately 3.5–4 hr over two sessions, and the 10 PART subtests repeated twice took approximately 2 hr over two sessions. Thus, to complete only two to three subtests, repeated twice in their current form using PART, the time would be approximately 6 min per test or 18 min for one repetition of dichotic FM and both SRM tests and 36 min for two repetitions. In its current form, if patients were able to come to the clinic 30 min prior to their appointment, they could foreseeably take the tests on a portable device in a quiet room with minimal supervision with the assistance of an intuitive user interface, such as that used with PART. However, with further testing using larger sample sizes, normative data could be collected and used to develop a brief screening tool. Adding three screening tests that could potentially take less than 15 min to complete in a quiet room on a portable device would allow additional tests, such as Dichotic FM and SRM, feasible to complete on the same day as a diagnostic hearing appointment. Future directions of this research will be to increase sample size to develop normative data for these binaural hearing subtests. Additionally, appropriate for the current COVID-19 pandemic, further testing will be conducted remotely to evaluate if performance is altered if testing is conducted at the patients' home when instructed to complete testing in a quiet room with minimal distractions.

In this small data set, there were some oddities in the SRM data. Although SRM showed variability across HI and NH listeners with similar pure-tone hearing or speech-in-noise ability, all NH and several HI participants in this experiment performed better than the normative data obtained by Lelo de Larrea-Mancera et al. (2020) for the spatially separated condition. Participants in this study also performed better than the participants in Gallun et al. (2018) and better than would be predicted (1.9-dB TMR better for colocated and 4.1-dB TMR better for spatially separated) based on the normative functions provided by Jakien and Gallun (2018). It should be noted that the progressive track utilized in this experiment had a ceiling of −10-dB TMR, which was met by several NH participants. Thus, while variability should be shown for the NH listeners (Kidd et al., 2008; Lutfi et al., 2018), performance by the NH group were limited by the methods chosen. Better performance on the spatially separated condition of the SRM may be due to increased attention and instruction by communication sciences and disorders students working in the lab, as opposed to relying on the instructions available in the PART application. Gallun et al. (2013) also described that individuals with good spatially separated thresholds tend to be at or near ceiling with thresholds between −8- and −10-dB TMR. Since a primary interest here is separating out HI listeners or those with poor speech-in-noise ability, the limitations of this test in showing variability for young NH listeners is outweighed by the ability to identify poor performers with an efficient testing procedure. Further testing with a larger sample size is needed to address these issues; however, participants evaluated at Western Washington University in comparison to other testing sites have consistently performed better on only the spatially separated SRM condition, compared to the other nine subtests evaluated using PART.

A larger sample size is also necessary to confirm these results and create normative ranges for a wider set of hearing losses and ages. While a few auditory profiles were observed in this small data set, evaluating only seven HI participants will limit the number of potential auditory profile patterns. However, despite the small *n* evaluated here, Dichotic FM and SRM appear to be two tests that could add information to current clinical protocols. Observing results from the other subtests, Spectral and Spectrotemporal Processing Ability and 2-kHz Noise Masking subtests, did not add any additional information to the current clinical diagnostic test battery. These tests evaluate frequency specificity and are all strongly correlated to pure-tone average as well as correlate with each other. Furthermore, tests of temporal processing and TFS (i.e., Diotic FM and Gap Detection) have not been shown to be strongly correlated with hearing loss as defined by the audiogram (Gallun et al., 2014; Spencer et al., 2016) and as observed in our study. Tests evaluating temporal processing were also highly correlated with each other in the current study, confirming good reliability within this small data set. Correlations across tests evaluating similar auditory ability (e.g., pure tones vs. QuickSIN, temporal task, spectral tasks) were more strongly correlated than any subtests correlated with 4FreqPTA or QuickSIN. Thus, the recommendation of adding dichotic FM and SRM will likely add to the current clinical protocol, potentially filling in gaps between specific auditory ability such as pure-tone hearing sensitivity and functional ability such as real-world performance in noisy environments such as the cocktail party problem.

The recommendations offered here are specifically focused on the patients whose auditory complaints are not in line with their performance on the current clinical diagnostic test battery. For example, patients seen by audiologists at Department of Veterans Affairs Medical Centers have been reporting large numbers of patients presenting with normal pure-tone hearing but complaints of difficulty hearing speech in noisy situations (Koerner et al., 2020). Gallun et al. (2012, 2016) provided data that may explain this trend, in which individuals with normal pure-tone hearing who were exposed to high-intensity blasts are likely to have difficulty on a range of auditory processing tests. Similarly, patients who have suffered concussions in nonmilitary settings are also likely to report auditory difficulties (Theodoroff et al., 2020), but audiological services are rarely provided, and when they are, the testing usually involves only pure-tone audiometry (Koerner et al., 2020). These studies show that patients with “normal” pure-tone hearing presenting with auditory complaints should be provided with further tests of auditory processing to better understand their complaints (Gallun et al., 2017). Such testing can put those patients at ease, confirming that they are outside of the normal range on particular auditory tasks that may translate to their functional difficulties experienced outside of the clinical setting. Koerner et al. (2020) also found that many of those with normal audiograms but auditory complaints appear to benefit from low-gain hearing aids. Such rehabilitation options, in addition to counseling, should be offered in combination with tests of auditory processing.

In summary, auditory processing tests that emphasize binaural hearing showed variability across HI participants and added information to auditory ability beyond a pure-tone and clinical speech-in-noise test. Specifically, dichotic FM and SRM evaluated on a portable platform is feasible to complete before or after a standard diagnostic test battery and may add information to patient's basic auditory ability profile. When patients perform well on a speech-in-noise test and have normal-to-near normal pure-tone hearing, or if their pure-tone results fail to match clinical speech-in-noise tests that are often restricted to monaural or diotic presentation, tests of binaural processing may be able to fill in the gaps. In counseling patients with functional auditory complaints, often these patients just need to be believed when they state difficulty with hearing in their everyday environments. Adding tests of binaural hearing may fill in the gaps between pure-tone testing and clinical speech-in-noise tests and will likely greatly reduce anxiety for patients with functional hearing complaints. Further testing is needed to turn dichotic FM and SRM into brief screening tests, but it is currently feasible to evaluate these binaural tests on a remote platform conducted in a quiet room with minimal instruction.

## Author Contributions

**Anna C. Diedesch:** Conceptualization (Lead), Data curation (Lead), Formal analysis (Lead), Funding acquisition (Equal), Investigation (Equal), Methodology (Lead), Project administration (Lead), Supervision (Lead), Visualization (Lead), Writing – original draft (Lead), Writing – review & editing (Equal). **S. J. Adelaide Bock:** Formal analysis (Supporting), Funding acquisition (Equal), Investigation (Equal), Visualization (Supporting). **Frederick J. Gallun:** Conceptualization (Supporting), Formal analysis (Supporting), Methodology (Supporting), Visualization (Supporting), Writing – original draft (Supporting), Writing – review & editing (Equal).

## References

[bib1] Bernstein, J. G. , Mehraei, G. , Shamma, S. , Gallun, F. J. , Theodoroff, S. M. , & Leek, M. R. (2013). Spectrotemporal modulation sensitivity as a predictor of speech intelligibility for hearing-impaired listeners. Journal of the American Academy of Audiology, 24(4), 293–306. https://doi.org/10.3766/jaaa.24.4.5 23636210 10.3766/jaaa.24.4.5 PMC3973426

[bib2] Bolia, R. S. , Nelson, W. T. , Ericson, M. A. , & Simpson, B. (2000). A speech corpus for multitalker communications research. The Journal of the Acoustical Society of America, 107(2), 1065–1066. https://doi.org/10.1121/1.428288 10687719 10.1121/1.428288

[bib3] Füllgrabe, C. , Moore, B. C. J. , & Stone, M. A. (2015). Age-group differences in speech identification despite matched audiometrically normal hearing: Contributions from auditory temporal processing and cognition. Frontiers in Aging Neuroscience, 6, 347. https://doi.org/10.3389/fnagi.2014.00347 25628563 10.3389/fnagi.2014.00347 PMC4292733

[bib4] Gallun, F. J. , Diedesch, A. C. , Kampel, S. D. , & Jakien, K. M. (2013). Independent impacts of age and hearing loss on spatial release in a complex auditory environment. Frontiers in Neuroscience, 7(7), 252–252. https://doi.org/10.3389/fnins.2013.00252 24391535 10.3389/fnins.2013.00252 PMC3870327

[bib5] Gallun, F. J. , Diedesch, A. C. , Kubli, L. R. , Walden, T. C. , Folmer, R. L. , Lewis, M. S. , McDermott, D. J. , Fausti, S. A. , & Leek, M. R. (2012). Performance on tests of central auditory processing by individuals exposed to high-intensity blasts. Journal of Rehabilitation Research and Development, 49(7), 1005–1025. https://doi.org/10.1682/JRRD.2012.03.0038 23341276 10.1682/jrrd.2012.03.0038

[bib6] Gallun, F. J. , Lewis, M. S. , Folmer, R. L. , Hutter, M. , Papesh, M. A. , Belding, H. , & Leek, M. R. (2016). Chronic effects of exposure to high-intensity blasts: Results of tests of central auditory processing. Journal of Rehabilitation Research and Development, 53(6), 705–720. https://doi.org/10.1682/JRRD.2014.12.0313

[bib7] Gallun, F. J. , McMillan, G. P. , Molis, M. R. , Kampel, S. D. , Dann, S. M. , & Konrad-Martin, D. L. (2014). Relating age and hearing loss to monaural, bilateral, and binaural temporal sensitivity. Frontiers in Neuroscience, 8(8), 172–172. https://doi.org/10.3389/fnins.2014.00172 25009458 10.3389/fnins.2014.00172 PMC4070059

[bib8] Gallun, F. J. , Papesh, M. A. , & Lewis, M. S. (2017). Hearing complaints among veterans following traumatic brain injury. Brain Injury, 31(9), 1183–1187. https://doi.org/10.1080/02699052.2016.1274781 28981349 10.1080/02699052.2016.1274781 PMC6417420

[bib9] Gallun, F. J. , Seitz, A. , Eddins, D. A. , Molis, M. R. , Stavropoulos, T. , Jakien, K. M. , Kampel, S. D. , Diedesch, A. C. , Hoover, E. C. , Bell, K. , Souza, P. E. , Sherman, M. , Calandruccio, L. , Xue, G. , Taleb, N. , Sebena, R. , & Srinivasan, N. (2018). Development and validation of Portable Automated Rapid Testing (PART) measures for auditory research. Proceedings of Meetings on Acoustics. Acoustical Society of America, 33(1), 050002. https://doi.org/10.1121/2.0000878 30627315 10.1121/2.0000878 PMC6322842

[bib10] Grose, J. H. , & Mamo, S. K. (2012). Frequency modulation detection as a measure of temporal processing: Age-related monaural and binaural effects. Hearing Research, 294(1–2), 49–54. https://doi.org/10.1016/j.heares.2012.09.007 23041187 10.1016/j.heares.2012.09.007 PMC3505233

[bib11] Hoover, E. C. , Kinney, B. N. , Bell, K. L. , Gallun, F. J. , & Eddins, D. A. (2019). A comparison of behavioral methods for indexing the auditory processing of temporal fine structure cues. Journal of Speech, Language, and Hearing Research, 62(6), 2018–2034. https://doi.org/10.1044/2019_JSLHR-H-18-0217 10.1044/2019_JSLHR-H-18-0217 PMC6808371 31145649

[bib12] Jakien, K. M. , & Gallun, F. J. (2018). Normative data for a rapid, automated test of spatial release from masking. American Journal of Audiology, 27(4), 529–538. https://doi.org/10.1044/2018_AJA-17-0069 30458523 10.1044/2018_AJA-17-0069 PMC6436452

[bib13] Kidd, G. , Mason, C. R. , Richards, V. M. , Gallun, F. J. , & Durlach, N. I. (2008). Informational masking. In W. A. Yost , A. N. Popper , & R. R. Fay (Eds.), Auditory perception of sound sources (pp. 143–189). Springer Science+Business Media. https://doi.org/10.1007/978-0-387-71305-2_6

[bib14] Koerner, T. K. , Papesh, M. A. , & Gallun, F. J. (2020). A questionnaire survey of current rehabilitation practices for adults with normal hearing sensitivity who experience auditory difficulties. American Journal of Audiology, 29(4), 738–761. https://doi.org/10.1044/2020_AJA-20-00027 32966118 10.1044/2020_AJA-20-00027

[bib15] Lelo de Larrea-Mancera, E. S. , Stavropoulos, T. , Hoover, E. C. , Eddins, D. A. , Gallun, F. J. , & Seitz, A. R. (2020). Portable Automated Rapid Testing (PART) for auditory assessment: Validation in a young adult normal-hearing population. The Journal of the Acoustical Society of America, 148(4), 1831–1851. https://doi.org/10.1121/10.0002108 33138479 10.1121/10.0002108 PMC7541091

[bib16] Lutfi, R. A. , Tan, A. Y. , & Lee, J. (2018). Modeling individual differences in cocktail party listening. Acta Acustica United With Acustica, 104(5), 926–929. https://doi.org/10.3813/AAA.919246

[bib17] Marrone, N. , Mason, C. R. , & Kidd, G., Jr. (2008). The effects of hearing loss and age on the benefit of spatial separation between multiple talkers in reverberant rooms. The Journal of the Acoustical Society of America, 124(5), 3064–3075. https://doi.org/10.1121/1.2980441 19045792 10.1121/1.2980441 PMC2736722

[bib18] Moore, B. C. (1987). Distribution of auditory-filter bandwidths at 2 kHz in young normal listeners. The Journal of the Acoustical Society of America, 81(5), 1633–1635. https://doi.org/10.1121/1.394518 3584698 10.1121/1.394518

[bib19] Patterson, R. D. (1976). Auditory filter shapes derived with noise stimuli. The Journal of the Acoustical Society of America, 59(3), 640–654. https://doi.org/10.1121/1.380914 1254791 10.1121/1.380914

[bib20] Smith, R. L. (1979). Adaptation, saturation, and physiological masking in single auditory-nerve fibers. The Journal of the Acoustical Society of America, 65(1), 166–178. https://doi.org/10.1121/1.382260 422812 10.1121/1.382260

[bib21] Spencer, N. J. , Hawley, M. L. , & Colburn, H. S. (2016). Relating interaural difference sensitivities for several parameters measured in normal-hearing and hearing-impaired listeners. The Journal of the Acoustical Society of America, 140(3), 1783–1799. https://doi.org/10.1121/1.4962444 27914394 10.1121/1.4962444 PMC5035301

[bib22] Strelcyk, O. , & Dau, T. (2009). Relations between frequency selectivity, temporal fine-structure processing, and speech reception in impaired hearing. The Journal of the Acoustical Society of America, 125(5), 3328–3345. https://doi.org/10.1121/1.3097469 19425674 10.1121/1.3097469

[bib23] Theodoroff, S. M. , Papesh, M. , Duffield, T. C. , Novak, M. , Gallun, F. J. , King, L. , Chesnutt, J. , Rockwood, R. , Palandri, M. , & Hullar, T. E. (2020). Concussion management guidelines neglect auditory symptoms. Clinical Journal of Sport Medicine. Advance online publication. https://doi.org/10.1097/JSM.0000000000000874 10.1097/JSM.0000000000000874 PMC7956904 32941367

[bib24] Theunissen, F. E. , & Elie, J. E. (2014). Neural processing of natural sounds. Nature Reviews Neuroscience, 15(6), 355–366. https://doi.org/10.1038/nrn3731 24840800 10.1038/nrn3731

[bib25] Theunissen, F. E. , Sen, K. , & Doupe, A. J. (2000). Spectral-temporal receptive fields of nonlinear auditory neurons obtained using natural sounds. Journal of Neuroscience, 20(6), 2315–2331. https://doi.org/10.1523/JNEUROSCI.20-06-02315.2000 10704507 10.1523/JNEUROSCI.20-06-02315.2000 PMC6772498

[bib26] Venezia, J. H. , Thurman, S. M. , Richards, V. M. , & Hickok, G. (2019). Hierarchy of speech-driven spectrotemporal receptive fields in human auditory cortex. NeuroImage, 186, 647–666. https://doi.org/10.1016/j.neuroimage.2018.11.049 30500424 10.1016/j.neuroimage.2018.11.049 PMC6338500

[bib27] Whiteford, K. L. , Kreft, H. A. , & Oxenham, A. J. (2017). Assessing the role of place and timing cues in coding frequency and amplitude modulation as a function of age. Journal of the Association for Research in Otolaryngology, 18(4), 619–633. https://doi.org/10.1007/s10162-017-0624-x 28429126 10.1007/s10162-017-0624-x PMC5532184

[bib28] Whiteford, K. L. , & Oxenham, A. J. (2015). Using individual differences to test the role of temporal and place cues in coding frequency modulation. The Journal of the Acoustical Society of America, 138(5), 3093–3104. https://doi.org/10.1121/1.4935018 26627783 10.1121/1.4935018 PMC4654737

